# LONG-REMI: An AI-Based Technological Application to Promote Healthy Mental Longevity Grounded in Reminiscence Therapy

**DOI:** 10.3390/ijerph19105997

**Published:** 2022-05-15

**Authors:** Àngela Nebot, Sara Domènech, Natália Albino-Pires, Francisco Mugica, Anass Benali, Xènia Porta, Oriol Nebot, Pedro M. Santos

**Affiliations:** 1 Soft Computing Research Group at Intelligent Data Science and Artificial Intelligence Research Center, Universitat Politènica de Catalunya, 08034 Barcelona, Spain; fmugica@cs.upc.edu (F.M.); anass.benali@gmail.com (A.B.); 2 Fundació Salut i Envelliment, Universitat Autònoma de Barcelona, 08041 Barcelona, Spain; sara.domenech@uab.cat (S.D.); xenia.porta@uab.cat (X.P.); 3 Escola Superior de Educação, Instituto Politécnico de Coimbra, 3030-329 Coimbra, Portugal; natalia.pires@gmail.com; 4 UX/UI Dessign Department, Universitat Oberta de Catalunya Barcelona, 08035 Barcelona, Spain; nebotoriolwork@gmail.com; 5 CINTESIS—Center for Health Technology and Services Research, Universidad de Lusófona Humanidades e Tecnologias, 1749-024 Lisboa, Portugal; p4087@ulusofona.pt

**Keywords:** reminiscence therapy, cognitive impairment, intangible cultural heritage, emotions recognition, face tracking, reinforcement learning

## Abstract

Reminiscence therapy (RT) consists of thinking about one’s own experiences through the presentation of memory-facilitating stimuli, and it has as its fundamental axis the activation of emotions. An innovative way of offering RT involves the use of technology-assisted applications, which must also satisfy the needs of the user. This study aimed to develop an AI-based computer application that recreates RT in a personalized way, meeting the characteristics of RT guided by a therapist or a caregiver. The material guiding RT focuses on intangible cultural heritage. The application incorporates facial expression analysis and reinforcement learning techniques, with the aim of identifying the user’s emotions and, with them, guiding the computer system that emulates RT dynamically and in real time. A pilot study was carried out at five senior centers in Barcelona and Portugal. The results obtained are very positive, showing high user satisfaction. Moreover, the results indicate that the high frequency of positive emotions increased in the participants at the end of the intervention, while the low frequencies of negative emotions were maintained at the end of the intervention.

## 1. Introduction

The increasing longevity of the population, resulting from the continuing decline in birth rates and increasing life expectancy, is transforming the shape of the age pyramid in the European Union, which will lead to a much older population structure [1]. The percentage of older people in relation to the total population will increase considerably over the next decades, when a large part of the baby boom generation reaches retirement age. This, in turn, will lead to an increased burden on working-age people, not only in terms of coping with the social expenditure required by the aging population, but also in terms of the intrafamily effort to care for their elders.

Furthermore, the longevity of the population brings a major challenge given that a significant proportion of older adults suffer from cognitive impairment and/or a significant decrease in memory, which alarmingly reduces their quality of life [2]. With this perspective on the table, it is particularly important to focus on the older people, with the aim of helping to make their lives at this stage active, rewarding, and fulfilling. In this sense, any action aimed at cognitive abilities, such as attention, memory, or concentration, is of particular relevance. A person with cognitive ability without impairment or with limited impairment has, in general, a more stimulating life, better performance, a healthier existence, and a higher predisposition to be an active member of society. It is, therefore, a matter of aging optimally. While mental inactivity may increase the risk of cognitive decline [3], engaging in cognitively stimulating activities may protect against cognitive decline in older age [4].

### 1.1. Reminiscence Therapy

Among the stimulation and activity programs aimed at the elderly, reminiscence therapy (RT) is the most effective in improving the quality of life of older people with cognitive impairment [5,6,7,8]. It consists in thinking about one’s own experiences through the presentation of memory-facilitating stimuli (photographs, images, etc.), using the past as a method to enjoy the communication of the present [5].

RT has proven to be a very powerful and effective cognitive neurorehabilitation technique. RT goes one step further than classical cognitive stimulation, because of the role played by emotions. Memories of events that reinforce the person’s identity usually evoke strong emotions [9]. It has been shown that people with severe cognitive impairments, despite cognitive deficits and despite losing their memory, keep their emotions alive until the end of life [5].

The development and evaluation of psychosocial interventions aimed at benefiting the elderly is a topic of growing interest. In this framework, cognitive stimulation therapies are recommended to maintain cognitive function, functionality, and quality of life of long-lived people [10]. Cognitive intervention is based on the concepts of neuroplasticity [11] and psychostimulation, and t is a highly individualized stimulation [12] adapted to the residual capacities of the person. Within the stimulation and activity programs, it is in RT that we find the most evidence [13]. RT is centered around shared themes and is often applied in long-term care [14]. Evidence for the efficacy of reminiscence interventions has accumulated over the past few decades. For older people with some degree of cognitive impairment, reminiscence can help improve communication [15], feelings of belonging [16], quality of life [17], mood, wellbeing, and life satisfaction [18]. Last but not least, it has positive effects on cognition [19]. Regarding cognition, a review of RT for dementia that included 22 randomized controlled trials concluded that there was high-quality evidence for a very small benefit associated with reminiscence at the end of treatment (standardized mean difference 0.11; 95% confidence interval 0.00 to 0.23; 14 studies; 1219 participants). Nine studies that included the Mini-Mental State Examination (MMSE) as a cognitive measure concluded that there was high-quality evidence for an improvement at the end of treatment (mean difference 1.87; 95% confidence interval 0.54 to 3.20; 437 participants). As a conclusion, RT had some positive effects on people with dementia in the cognitive domain [5].

Recently, very successful innovative reminiscence interventions have been developed on specific topics of general interest for a large part of the population, such as soccer [16]. However, this type of therapy is expensive and difficult to implement. It requires specialized personnel for the preparation of the necessary material and the follow-up of the RT session. This leads to this type of therapy being performed without continuity, mainly focusing on patients with dementia [8]. However, the benefits of RT may also be useful for healthy older adults [20]. In this sense, we believe that having an AI-based application to facilitate RT to older people with or without cognitive impairment would lead to an improvement in their quality of life.

### 1.2. State of the Art

We can find a substantial number of software tools on the market, generally apps, that deals with some aspects of memory loss and cognitive diminishing. A large number of these focus on Alzheimer’s disease, as it is the most common cause of dementia, accounting for an estimated 60% to 80% of cases [21]. In order to analyze their objectives and characteristics, we carried out a study of the most relevant ones found in the most widely used repositories, as well as in repositories in the field of health, such as Play Store, App Store, TIC Salut, CREA, ACTO Dementia, and CEAPAT. The study also includes an analysis of the most recent literature in the field.

There are different types of applications depending on who they are intended for: people with cognitive impairment, their caregivers, or medical and care professionals. Applications for caregivers offer advice and tools to improve attention, care and safety of the patient, and location of the person, as well as extensive and updated information on different resources related to Alzheimer’s and other dementias. Applications for people with cognitive impairment can be classified into two main groups, those that provide cognitive stimulation and those for orientation and reminders of daily activities. Apps for medical and care professionals are basically focused on early diagnosis. It is worth mentioning that the apps aimed at caregivers are more numerous than those aimed at professionals.

A systematic review of the literature reveals that the existence different studies that analyzed the availability, content, characteristics, and quality of different apps focused on cognitive impairment. A good example of this can be found in [22], where the authors studied the apps available for people suffering from cognitive impairment or Alzheimer’s disease, with the limitation that they are available only in English. The authors concluded that available apps may not meet complex needs and may be difficult to use given the potential reduction in communication ability associated with cognitive impairment. Therefore, they consider it necessary that high-quality apps be developed and rigorously evaluated to determine their feasibility and effectiveness. Along the same lines, in [23], the benefits and limitations of the use of apps in the management and assessment of mild to moderate cognitive defects were analyzed. In this work, it was concluded that apps are mostly useful to support people with cognitive limitations in daily life tasks, especially, in the early diagnosis of this disorder. However, it is considered necessary that new developments of such apps meet the specific needs of older adults. They consider that there is a need to develop apps that are multifunctional, sensitive to the culture of minority populations, easy to read and use, and, when necessary and appropriate, facilitate information sharing with families, providers, and caregivers. Some work can also be found in the literature looking at the use of technology more particular to RT [24,25,26,27]. These studies, which contained many interesting ideas to consider regarding the interaction of people with cognitive impairment with a technological device in a broad sense (tv, video, computer, cell phone, etc.), basically presented technological resources for the support of RT content. We did not find in the literature any technological application/tool that aims to carry out RT, i.e., to guide users through a set of contents on the basis of their reaction to them. The main objective of this study was to provide older people with a tool that allows them to carry out RT on a regular, individualized, dynamic basis, adapted to their level of cognitive impairment. The continued application of this therapy can help make their life at this stage active, rewarding, and fulfilling.

Focused on this goal, we were interested in a person-centered approach, analyzing in more depth existing applications aimed at people with cognitive impairment and not so much at their caregivers or medical and care professionals. Of all the applications found and analyzed, we present a set of nine tools that we consider relevant, regarding the ratings and opinions of users, as well as the organizations that support them:

- AlzhUp: Personal memory bank to which the caregiver and family members can contribute. It also incorporates aerobic, calculation, memory, or relaxation games and exercises [28].
- Memory Box: Aims to stimulate long-term memory by storing photographs and audios of everyday experiences while also encouraging conversations and exchanges between an elderly person and the caregiver or family member [29].
- Refresh My Memory—An application to help remember objects and their location, as well as people in the user’s environment [30].
- YoTeCuido Alzheimer: Support application for caregivers and patients. It provides information about Alzheimer’s disease and allows establishing routines and exercises. The main novelty of its contents compared to other available apps is that they are based on the experience of caregivers and family members, as well as patients and professional caregivers [31].
- Backup Memory: Creates a memory album. The app reminds users of their relationship with each family member by showing them photos and videos of past experiences they shared with their family member [32].
- Imentia: Cognitive stimulation based on a series of interactive exercises that train the different cognitive areas: memory, orientation, language, attention, reasoning, comprehension, etc. Each work area has different levels of complexity [33].
- NoMeOlvides: Application made up of a series of mini-games classified in different categories, such as shapes, colors, addition and subtraction, animals, and objects. It uses an assistant that guides the user through the application [34].
- Andzheimer: Through different graphic exercises divided into their corresponding cognitive areas, it tries to enhance memory, attention, language, executive functions, etc. [35].
- Stimulus: It is based on a series of interactive exercises that train the different cognitive processes, attention, perception, working memory, long-term memory, calculation, reasoning, executive functions, etc. [36].

It is also interesting to mention the work of Caros et al., which created a reminiscence chatbot that uses artificial intelligence (AI) technology to drive communication through the use of a combination of photos and questions directed at people over 60 years of age with mild cognitive impairment. They designed a usability study in which users interacted with the system, with help [37]. In order for users to be able to interact with technology in the easiest and most intuitive way possible, it must meet the user’s needs; therefore, usability testing is essential [37,38]. Although this abovementioned work developed a chatbot to generate a conversation with the user from a photo with the aim of generating reminiscence, this application does not evaluate whether or not reminiscence occurs in the user nor does it generate a specific conversation derived from the level of reminiscence detected.

As a conclusion of the state of the art of the research carried out, no applications have been found that allow carrying out an RT that meets the characteristics of RT guided by a therapist or a caregiver. Of the nine applications mentioned, AlzhUp and Memory Box are the closest to our work. These have a common functionality to our proposal that they call “memory bank”. This functionality consists of encouraging the participation of various family members to include and validate videos or photographs of shared experiences in the application. This point is also an important aspect of our research since RT is carried out on events that have influenced the life of the elderly person, at both a personal and a family level, as well as at the level of the public environment of the country and the world in general. Therefore, the creation of a database, consisting of all kinds of resources, including photographs, videos, voiceovers, songs, and news, as wide as possible, will be of great interest and relevance to effectively carry out RT. However, in this research, we do not rely on the personal material of the user; instead, we focus on the theme of intangible cultural heritage.

In this paper, we present research whose main objective was the development of an AI-based application that recreates personalized RT, framed within the theme of intangible cultural heritage, to help to improve the quality of life of long-lived people. We believe that an application that facilitates RT in individual sessions, which adapts dynamically and in real time to the emotions of the elderly person using it, will have a relevant impact on that person’s cognitive stimulation or cognitive maintenance.

Section 2 describes the main aspects of the research performed over the course of the project in order to address this challenge. Section 3 presents and discusses the results of the interventions carried out in the groups of the elderly (subjects without cognitive impairment; subjects with cognitive impairment) in centers in both Spain and Portugal. Lastly, conclusions are highlighted.

## 2. Materials and Methods

This section pays deep attention to the development of the AI-based application, as well as the description of the pilot study, the participants involved, the clinical evaluation, and the outcome variables.

### 2.1. Intangible Cultural Heritage Material

While some research investigated reminiscence from topics such as music [39], crafts [40], art [41], and soccer [16], the use of intangible cultural heritage as a topic for reminiscence therapy has not yet been explored.

The various manifestations of intangible cultural heritage, particularly oral literature and traditional music or dance, have an important function within communities that contributes to the construction of individual and collective identity, in addition to being part of the cultural heritage of the community [42].

A relevant part of the work in this research project was the collection, analysis, selection, and adaptation of material from the intangible cultural heritage of Spain and Portugal. The selection of intangible heritage manifestations to be used in stimulation sessions as an inductor was performed considering the origin, age, and literacy level of the participants. We resorted to open-access databases of intangible heritage in each region and to open-access content extracted from radio and television. We focused on material from the period 1930–1970.

### 2.2. AI-Based Application Development

The development of the application entailed the design and implementation of the different elements constituting it as listed and described below:

- An internal storage structure for the material that makes up the intangible cultural heritage, as well as patient information. With patient information, we refer both to the characteristics of each patient (age, place of birth, place where they live, level of cognitive impairment, can or cannot read) and to the knowledge that the application acquires as the user progresses in RT (what type of activities and cultural heritage causes reminiscence reactions, what type of cultural heritage do they like more, i.e., tongue twisters, songs, proverbs, or dances).
- A specific technique for facial emotion recognition focusing on the analysis of the expression of the mouth and eyes. Our approach is framed in the area of face tracking. On the basis of the identified shape of the mouth and the degree of eye opening, it defines a smile index in the range [0, 1]. This index indicates confidence in the presence of a smile. For example, a value equal to or greater than 0.7 indicates the likelihood that a person is smiling is very high.
- An algorithm for optimal pathfinding maximizing the possibility of reminiscence dynamically, depending on the emotions of the person performing the RT. This algorithm aims to guide the application by the cultural heritage introduced in the internal storage structure considering the person who is using the system, their personal characteristics, and the reminiscences detected throughout the therapy session. Therefore, the program learns from the user’s reactions, in order to provide the best therapeutic stimuli at each moment.
- A large set of activities of the different representations of intangible cultural heritage, i.e., proverbs, tongue twisters, songs, and dances.

It should be noted that all the activities were designed, analyzed, discussed, and agreed upon all the researchers of the project, such that all the relevant aspects of the different areas (simple and accessible technology, adequate access to intangible cultural heritage and adaptation to the diversity of the older adults) were considered. AI has the potential to transform struggling healthcare systems through new therapies, as well as enable synergies between technologists and healthcare professionals. In relation to LONG-REMI, we could not find any application that performs TR and uses AI techniques to offer the user different content on the basis of their reaction or that assesses the participant’s emotions.

### 2.3. Face Emotion Recognition

**Figure 1:**
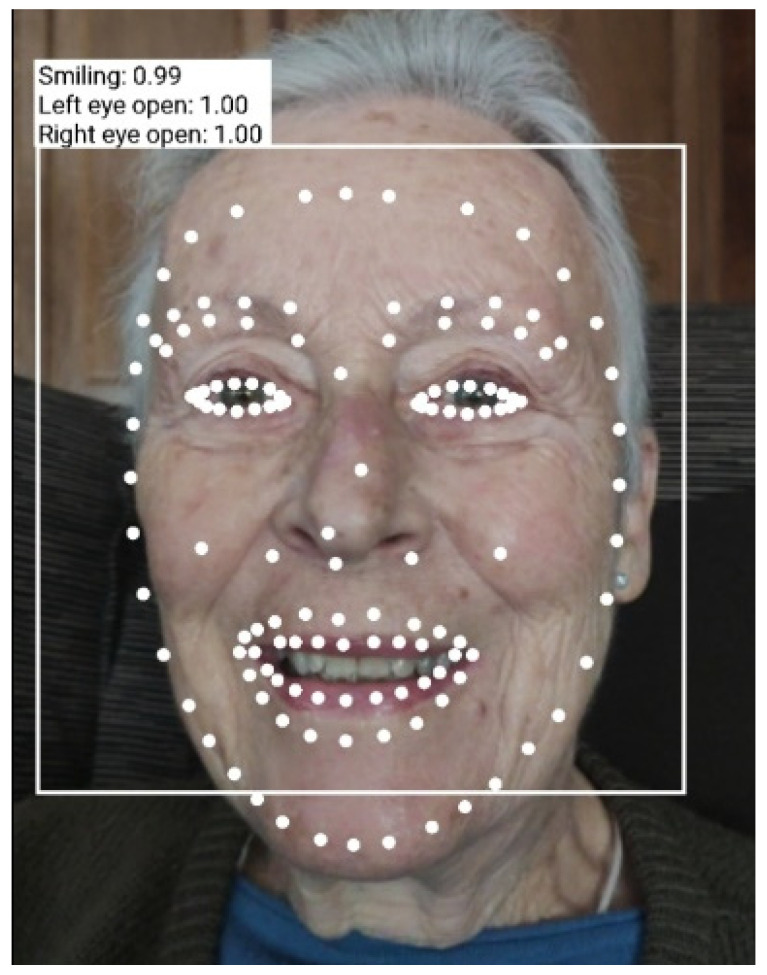
Example of identification of a face with the corresponding smile index. The smile index in this case is of 0.99, meaning that the person is clearly smiling.

The technique developed is able to identify key facial features (in our case, mouth, eyes, and nose), and get the contours of detected faces in real time. It is based on ML Kit SDK [43]. The application records an image of the user every second and automatically identifies the face and the contours of the eyes, mouth, nose, and eyebrows, as shown in Figure 1.

From the mouth and eye contours, the algorithm recognizes facial expressions. Tensorflow Lite models (neural networks and deep learning) are used. Lite models are used for low-power mobile devices. The classifier based on deep learning determines if a certain facial characteristic is present (i.e., in our case, mouth and eyes open). The degree of match of the classifier indicates the confidence that the facial characteristic we are looking for appears in the image. The confidence is expressed as an index in the range [0, 1]. These classifications rely upon landmark detection. A landmark is a point of interest within a face. In our case, the landmarks computed and analyzed are the left eye, right eye, nose, and mouth.

This technique was incorporated in the framework of the reminiscence application, where a thorough validation of its good performance was carried out in the environment of use, i.e., on a tablet (with Android operating system), with the same characteristics as those to be used in the different senior centers. On the other hand, smile detection tests were performed with different users in order to validate the accuracy of the program when applied to different persons, with different characteristics.

It should be noted that it is very important that the smile detection technique has a high level of sensitivity (as well as high accuracy) since it will be used in elderly people with different degrees of cognitive impairment, which often involves facial stiffness and expressionlessness. The test results were satisfactory when applied to older people.

### 2.4. Path Finding Algorithm

The algorithm designed to guide the RT works as a fully connected graph for each category that constitutes the intangible cultural heritage material. Materials are selected on the basis of the weights assigned to each node of the graph. Thus is essentially a weighted sampling without replacement of the materials, where the weights evolve as the elderly person uses the application and reacts to the materials presented to them.

All materials start with a weight of 1 for the set of materials of a category and a user. We analyze the users’ degree of smiling every second and obtain a probability in the range [0, 1]. If this probability is greater than 0.33, then the weight of that material is updated following Equation (1).

(1) $${\mathrm{weight}}_{\mathrm{new}}=\left(1+{\mathrm{weight}}_{\mathrm{prev}}\right)\cdot \left(1+P\left(\mathrm{smile}\right)\right).$$

First, the weight of a material is updated by adding 1 to the previous weight. Second, the new weight is scaled proportionally by a certain percentage in the range [1.33, 2.0], i.e., by an additional 33.33% to 100%.

The weights are also modified when the users answer the different questions that appear in some of the application’s activities. If users indicate that they do not like or do not know the material, the weight of this material is set to 0.1. If users respond that they do like the material shown, then the weight is modified as indicated in Equation (2).

(2) $${\mathrm{weight}}_{\mathrm{new}}=1+{\mathrm{weight}}_{\mathrm{prev}}+\frac{N}{8},$$

where N is the amount of material in that category. Note, that the application has four categories. i.e., songs, dances, proverbs, and tongue twisters. The idea is to increase the weight proportionally to the amount of material available. Whether the user likes or smiles at a material among a large number of possible materials in a category is more relevant than whether the user likes a material among a small number of materials in that category. If there is little material in a certain category, it will end up being displayed anyway as there is not much variety available. Conversely, if there is a lot of material in that category, some of it will not have a chance to be shown and, therefore, there will be subjected to competition among the available material.

The current algorithm is based on reinforcement learning, in the sense that a material that provides a positive reaction to users is rewarded, while a material that provides a nonreaction or negative reaction to users is penalized.

All the constants defined in this algorithm were chosen heuristically, and it is expected that the continued use of the application by a sufficiently large number of elderly people from different Spanish and Portuguese centers will provide the necessary information to derive the algorithm in the form of a recommender. In this way, when sufficient metadata are available, it will be possible to choose the next material to be shown to the user based on its similarity, e.g., between user profiles. This first version of the application will make it possible to acquire information on its use by the elderly and, from there, to improve the algorithm with the available information.

### 2.5. App Activities

Listed below are some of the activities designed for each of the intangible cultural heritage manifestations and available in the application:

Songs

- Audio/video is presented showing one or more people singing a traditional song. The user listens to the song.
- Users are asked several questions about the song they have heard: Do you know the song? Do you remember who sang it? Who did you sing it with or listen to it with? Do you usually sing it? The user selects an answer from the displayed list.
- A list of music topics is displayed. Users are asked which topic(s) they like the most. The user selects an answer from the displayed list.

Dances

- The activities associated with the dances are the same as those discussed for the songs, adapting the questions to be consistent.

Tongue Twisters

- The activities associated with tongue twisters are the same as those listed for songs. These activities are presented by voice and also by text that appears on the screen.

Proverbs

- The whole proverb is shown. The user is asked several questions: Have you heard it before? Do you remember who said it? Do you usually use it?. The user selects an answer from the displayed list.
- The first part of the proverb is shown. The user must finish the proverb by selecting the words that appear unordered on the screen or by typing the text from a keyboard.
- The second part of the proverb is displayed. The user must complete the proverb by selecting the words that appear unordered on the screen or by typing the text from a keyboard.
- A list of *n* proverbs (default *n* = 3) is displayed on the left-hand side of the screen, and a list of their unordered meanings is displayed on the right-hand side. The user has to match each proverb with its meaning.
- A list of the first part of *n* proverbs (default *n* = 3) is displayed on the left-hand side of the screen, and a list of the second part of the previous *n* proverbs is displayed unordered on the right-hand side of the screen. The user must join the two parts of each proverb.
- A proverb and a list of categories are displayed. The user must select the category to which the proverb belongs.
- A proverb is shown leaving one or more empty words. A list of words is also displayed. The user must complete the proverb by selecting the correct words.
- A proverb is displayed, and the system asks the user to read it. The user’s voice is recorded and used in case the proverb comes up again in any of the subsequent RT sessions.

**Figure 2:**
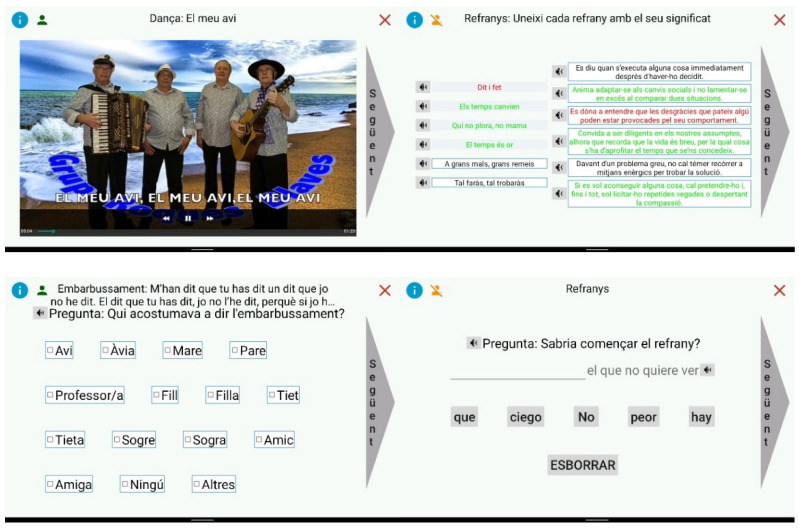
Example of screenshots of the application (in Catalan). The application contains material in Catalan and Portuguese as it is intended for citizens of both countries.

Figure 2 shows some screenshots of the application to give a general idea about how the activities look.

The process of defining the activities and designing the application environment was dynamic and iterative, such that the format of the screens and the presentation of the different activities within the platform were refined, with the aim of achieving an intuitive and simple interaction between the application and the user, considering all possible limitations of the users.

All the information that appears on the screen can be read by the computer itself so that those users who cannot read can perform the RT without any problem. To this end, all the phrases that appear on the screen will have a loudspeaker symbol next to them, to indicate to the user that they can hear the corresponding phrase.

Another relevant aspect is that some of the activities may have different degrees of difficulty. Depending on the educational level and the degree of cognitive impairment of the elderly person, the application will adapt the difficulty of these activities.

### 2.6. Pilot Study and Participants

A prospective multicenter observational pilot study was conducted in three senior centers in Barcelona (Spain) and two senior centers in Portugal, in which the applicability of the developed application for elderly people of a reminiscence program based on intangible cultural heritage was evaluated.

The centers recruited as participants in the study users who met the inclusion criteria and who gave their consent (or caregiver or legal representative). The study included people aged 65 years or older, with no cognitive impairment, with mild cognitive impairment and with mild and/or moderate cognitive impairment, equivalent to Global Deterioration Scale (GDS) scores of 3, 4, and 5, respectively, according to Reisberg’s Global Deterioration Scale [44], who attended the centers and were not required to be able to read and write. The exclusion criteria were having severe mental, sensory, behavioral and/or cognitive alterations that could interfere with the intervention.

Forty-two participants were included from three elderly centers without cognitive impairment (*n* = 21) and two day centers with cognitive impairment (*n* = 21) from Spain and Portugal. Subjects had a mean age of 79.21 ± 7.22 (66–94); 30 were female (71.4%), and 29 had a low level of education (69.04%), including two subjects who could not read and write.

The main demographic characteristics of the subjects according to the groups are given in Table 1. For subjects with cognitive impairment, the Mini-Mental State Examination (MMSE) of Folstein [45] was collected as a clinical variable (Table 1). Subjects completed an average of 3.59 ± 0.40 sessions. Of the total sample, 22 subjects (52.38%) performed the tasks with a high level of difficulty. While older people without cognitive impairment performed the activities independently, people with cognitive impairment needed help in performing the activities.

### 2.7. Intervention Program

The app has activities designed for each expression of intangible cultural heritage selected: proverbs, tongue twisters, songs, and dances. The activities can be adapted to the users’ level of cognitive impairment. The program has more than 500 stimuli. The information displayed on the screen can be read by the tablet itself. Older adult volunteers were involved in the research from the beginning, in terms of design and the collection and selection of content. The AI-based application allows elderly people to carry out RT using intangible cultural heritage on a regular basis, individualized and adapted to their level of difficulty and preferences through a tablet. The program was previously validated with older adults of different educational and cognitive levels.

Individual weekly 30 min sessions were held for four consecutive weeks using the application. Given the applied nature of the work and with the main objective being the development of an AI-based application that recreates personalized RT, we did not contemplate creating a control group. In each of the centers, a specialized psychologist, previously trained in the program, supervised the sessions, adjusting the level of difficulty of the tasks to the degree of cognitive impairment of each user and educational level.

### 2.8. Clinical Evaluation and Outcome Variables

Before starting the reminiscence program, the participants included in the study were evaluated by two independent professionals (psychologists) in an initial visit. Demographic variables (age, gender, educational level, and place of birth) and clinical variables (PANAS) were collected. The Positive Affect and Negative Affect Scale (PANAS) is composed of 20 items describing positive or negative emotions, 10 of them positive and 10 of them negative. Each item is answered by means of a Likert-type ordinal scale with five response options (not at all, very little, somewhat, quite a lot, and very much). The lowest score that can be obtained is 20 and the highest is 100. The scale is composed of two subscales with each one referring to the type of emotions (positive and negative), where high scores in each of the subscales suggest a high presence of positive or negative emotions in the subject [46].

After completion of the reminiscence program, participants were reevaluated by the same professionals (psychologists), and the PANAS was readministered. The main study variables were usability and participant satisfaction collected at the end of the intervention, assessed with the VAS scale (visual analog scale). The VAS scale allows measuring the intensity described by the patient with respect to pain. In this case, this scale was used to measure usability and satisfaction. It consists of a horizontal line of 10 cm, at the ends of which are the extreme expressions from 1 = “not at all” to 10 = “very much”. On the left side is located the absence or lower intensity and on the right side is located higher intensity. The participant is asked to mark on the line the point that indicates the intensity measured with a millimeter ruler [47]. Usability was assessed with the following question: Did you find it easy to complete the reminiscence program? The score of the degree of difficulty of the sessions was measured on a scale of 1 to 10. Satisfaction was assessed with the following question: What is your level of satisfaction with the reminiscence program? The score of the degree of satisfaction with the sessions was measured on a scale of 1 to 10.

The perception of user experience was also assessed using the CSUQ [48]. The Computer System Usability Questionnaire (CSUQ) is a widely used questionnaire to measure the perception of user experience. The Spanish adapted version was used [49], with a seven-point Likert response scale (1 = strongly disagree; 7 = strongly agree), with higher scores indicating better perception. It consisted of 16 items: 1–6 (system use), 7–12 (information quality), 13–15 (interface quality), and 16 (overall estimation). Secondary outcome measures included the perception of positive and negative emotions of the participants, assessed from the Spanish validated version of the PANAS of Watson et al. [45,50].

## 3. Results and Discussion

Statistical analysis was performed with SPSS 21 software [51]. Baseline and follow-up clinical characteristics were expressed as the mean ± standard deviation. We used nonparametric tests for hypothesis testing, specifically the Wilcoxon test, to analyze pre- and post-intervention scores. Statistical significance was set at *p* < 0.05.

Table 2 shows the mean scores of the VAS usability (1–10) and satisfaction (1–10) scales, as well as the CSUQ (1–7), for the two study groups.

The results show a high score in usability and satisfaction in both groups, older people without and with cognitive impairment, with average scores of 7.86 ± 1.98 (usability) and 8.43 ± 1.59 (satisfaction). The satisfaction of the users’ perception with the experience in relation to the usefulness of the system, quality of information, and quality of the interface was also very high, with an overall mean CSUQ score for all subjects of 6.29 ± 0.64. The mean scores for the three subfactors were 6.09 ± 0.99, 6.04 ± 0.93, and 6.22 ± 0.87, indicating high satisfaction with the users’ perception of system utility, information quality, and interface quality, respectively.

Thus, these results suggest that this prototype is applicable to older people, both without cognitive impairment and with mild cognitive impairment and mild and moderate dementia. The technological application allows the participation of illiterate subjects and can be used in older people both with and without cognitive impairment.

In relation to the pre- and post-intervention scores, we found statistically significant differences in the PANAS positive affect subscale, but not in the PANAS negative affect subscale. Table 3 shows the pre- and post-intervention scores on the PANAS positive and negative affect subscales for both groups of subjects.

The frequency of positive emotions in the participants evaluated from the PANAS positive affect subscale increased significantly at the end of the intervention in relation to the beginning, indicating an increase in the positive feelings or emotions evaluated. In relation to the frequency of negative emotions in the participants evaluated from the PANAS negative affect subscale, no significant differences were found at the end of the intervention in relation to the beginning. The explanation is that neither in the initial nor in the final evaluation did the subjects feel negative emotions while they were using the application.

Thus, we focused on usability and satisfaction, highly perceived by the participants. As a conclusion, this first prototype of the LONG-REMI technological application can be used in older people with and without cognitive impairment. Moreover, this research is a first approach to guarantee that our application has been conducted in accordance with fundamental ethical principles of respect for persons: beneficence, nonmaleficence, and justice [52].

The absence of a control group limits the possibility of drawing consistent conclusions about the effectiveness of treatment. However, the experience described seems to show that the developed application is a useful tool to treat the cognitive and emotional deficits of participants. Follow-up studies will be necessary to elucidate whether the improvements observed are maintained in the medium and long term.

**Figure 3:**
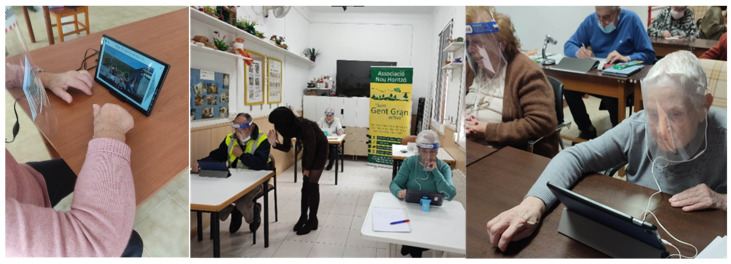
Volunteers during a LONG-REMI session.

In addition to the evaluation based on the results of tests, questionnaires, and tools in the psychological and psychiatric fields, as well as the statistical analysis of these, we consider it important to mention another type of results, more empirical, based on the perception and analysis of the physical reaction of the elderly when using the application. In this sense, in all the sessions and in all the centers where the intervention was carried out, many of the participants had clear physical reactions that showed their satisfaction, joy, and/or wellbeing during the activity they were carrying out. Examples of this were tapping their hands on the table to follow the rhythm of the music, moving their feet when they were visualizing a dance, or talking when they were performing an activity related to tongue twisters or proverbs. Figure 3 present photos of the sessions in different centers to show how the volunteers used the application.

## 4. Conclusions

Reminiscence therapy (RT) emerged in the field of patients with dementia with cognitive impairment such as Alzheimer’s disease. It has been found that RT has more evidence of benefits for the patient within the stimulation and activity programs. The results of cognitive improvement and reinforcement confirm that RT is a beneficial and positive tool not only for patients but also for all long-lived people, helping to strengthen and maintain their cognitive abilities. However, this type of therapy is expensive and difficult to implement. This leads to this type of therapy being performed on an ad hoc basis only for patients with dementia, without continuity. In this research, an AI-based application that facilitates RT for older adults with or without cognitive impairment was developed.

The application incorporates techniques in the areas facial emotion recognition and reinforcement learning to evaluate the user’s emotions and, with them, to dynamically guide in real time a computer system to perform RTs. This application allows RT to be used in a generalized way by any citizen, adapted to individual sessions. On the other hand, to the best of our knowledge, there is currently no application such as the one developed.

To date, the application has been evaluated through five pilot studies conducted in Spain and Portugal. The results show, with statistical validation, a high satisfaction in the users’ perception of the usability of the system, the quality of the information, and the quality of the interface. Moreover, the results indicate that the high frequency of positive emotions increased in the participants at the end of the intervention. On the other hand, the low frequencies of negative emotions were maintained at the end of the intervention.

As future work, we plan to incorporate into the application all the potential improvements detected in the interventions carried out in the five centers for the elderly. Due to the interest generated by the application in other territories of Spain and Portugal, we intend to expand the material of the intangible cultural heritage and offer a new version of the application to all those senior centers interested in using it free of charge. A randomized controlled study with a longer follow-up time is needed, and we expect to do it in the near future, to evaluate the efficacy of this tool for the cognitive and emotional aspects of participants.

## Figures and Tables

**Table 1:** Demographic characteristics of the subjects. M ± SD indicates the mean ± standard deviation. Education level (low: no studies or primary studies; medium: high school or equivalent; high: university studies or higher).

	People without Cognitive Impairment (*n* = 21)	People with Cognitive Impairment (*n* = 21)
Age M ± SD (ranges)	76.67 ± 6.55 (66–87)	81.76 ± 7.08 (68–94)
Gender % women	66.2 (14 participants)	76.19 (16 participants)
Education level		100 (21 participants)
Low	38.1 (8 participants)	
Medium	47.6 (10 participants)	
High	14.3 (3 participants)	
MMSE M ± SD (ranges)		22.60 ± 5.48 (13–30)

**Table 2:** Usability (1–10) and satisfaction (1–10) scales, as well as CSUQ (1–7).

	People without Cognitive Impairment (*n* = 21)	People with Cognitive Impairment (*n* = 21)
Usability	8.29 ± 1.59	7.43 ± 2.38
Satisfaction	8.57 ± 1.78	8.29 ± 1.42
CSUQ		
Overall score	6.95 ± 0.22	5.64 ± 1.06
System utility	6.35 ± 0.97	5.83 ± 1.02
Information quality	6.04 ± 1.15	6.04 ± 0.72
Interface quality	6.71 ± 0.67	5.74 ± 1.07

**Table 3:** PANAS of positive and negative affect before and at the end of the intervention for both groups of subjects. Bold values indicate statistical significance (*p* < 0.05).

PANAS	Pre Intervention	Post Intervention	*p*-Value
People without cognitive impairment			
Positive affect subscale	31.81 ± 8.32	40.52 ± 10.17	0.001
Negative affect subscale	11.76 ± 2.32	11.62 ± 2.54	0.775
People with cognitive impairment			
Positive affect subscale	24.29 ± 8.39	34.57 ± 9.12	0.000
Negative affect subscale	12.24 ± 3.42	12.33 ± 3.32	0.975

## Data Availability

Not applicable.
